# Association between polymorphisms in *CXCR2* gene and preeclampsia

**DOI:** 10.1002/mgg3.578

**Published:** 2019-02-03

**Authors:** Hongqin Chen, Yanping Zhang, Li Dai, Yaping Song, Yanyun Wang, Bin Zhou, Rong Zhou

**Affiliations:** ^1^ Department of Obstetrics and Gynecology West China Second University Hospital, Sichuan University, Key Laboratory of Birth Defects and Related Diseases of Women and Children (Sichuan University) of Ministry of Education Chengdu Sichuan PR China; ^2^ Laboratory of Molecular and Translational Medicine West China Second University Hospital, Sichuan University, Key Laboratory of Birth Defects and Related Diseases of Women and Children (Sichuan University) of Ministry of Education Chengdu Sichuan PR China

**Keywords:** CXCR2, polymorphisms, preeclampsia

## Abstract

**Background:**

Preeclampsia is a serious pregnancy‐specific syndrome with incompletely understood pathogenesis. Previous study has demonstrated that the decreased CXCR2 in preeclamptic placentas may contribute to the development of preeclampsia. The role of single nucleotide polymorphisms (SNPs) of CXCR2 gene in the pathogenesis of preeclampsia remains largely unexplored. Thus, we aimed to investigate the association between polymorphisms of CXCR2 gene and preeclampsia in Han Chinese women.

**Methods:**

Totally 481 pregnant women, including 243 controls and 238 patients with preeclampsia were recruited. The rs1126579 and rs2230054 polymorphisms in CXCR2 gene were tested using polymerase chain reaction‐restriction fragment length polymorphism method.

**Results:**

Significantly increased risk of preeclampsia was observed in the rs1126579 CC or TC/CC genotypes when compared with TT genotype (CC vs. TT: odss ratio [OR] = 2.11, 95% confidence interval [CI] = 1.18–3.76, *p* = 0.039; TC/CC vs. TT: OR = 1.89, 95% CI = 1.29–2.78, *p* = 0.001). Markedly higher risk of preeclampsia was found to be associated with rs1126579 TC genotype (TC vs. TT/CC: OR = 1.48, 95% CI = 1.04–2.12, *p* = 0.031). After stratification analysis, the different distribution of TC/CC genotypes was particularly significant in the severe preeclampsia group (OR = 2.15, 95% CI = 1.42–3.24, *p* < 0.01), the early‐onset severe preeclampsia group (OR = 1.97, 95% CI = 1.14–3.42, *p* = 0.013), and the late‐onset severe preeclampsia group (OR = 2.29, 95% CI = 1.39–3.78, *p* < 0.01). Besides, TC genotype carriers had a 1.55 fold increased risk of severe preeclampsia (95% CI = 1.06–2.27, *p* = 0.022) and a 1.80 fold increased risk of late onset severe preeclampsia (95% CI = 1.14–2.83, *p* = 0.01) than those of TT/CC genotype carriers.

**Conclusions:**

Our study suggests a genetic association between rs1126579 polymorphism in CXCR2 gene and increased risk of preeclampsia. These data provide a new clue for future investigation.

## 1 INTRODUCTION

Preeclampsia is a multisystem pregnancy‐specific disease, affecting 5%–8% of pregnancies (Gathiram & Moodley, 2016). It is a major cause of perinatal morbidity and mortality worldwide. Moreover, women suffered preeclampsia and their offspring showed increased risk for cardiovascular events, metabolic and mental disorders in the future (Souza et al., 2013). The causes and pathogenic mechanisms of preeclampsia remain poorly defined and thereby no effective method for prevention and treatment is available. Genetic predisposition is thought to be an important etiological factor. Single nucleotide polymorphisms (SNPs) in genes have been discussed, and various genes were found to be associated with the risk of preeclampsia (Giannakou, Evangelou, & Papatheodorou, 2018). It is likely that multiple genes or SNPs may contribute to the development of preeclampsia (Giannakou et al., 2018). To fully elucidate the role of genetic factors in the pathogenesis of preeclampsia, more genetic researches are needed.

Chemokines exert various pathophysiological functions via binding to their receptors. The chemokine superfamily is categorized into four subgroups based on the positions of conserved cysteine amino acids in the N‐terminal region (CXC, CC, CX3C and C) (Vandercappellen, Van Damme, & Struyf, 2008). CXCR2 is the receptor of the Glu‐Leu‐Arg+ (ELR+) CXC chemokines, including CXCL1‐3 and CXCL5‐8 (Vandercappellen, Van Damme, & Struyf, 2008). Importantly, CXCR2 plays critical roles in inflammation (Boro & Balaji, 2017), immunity (Kang et al., 2016), oxidative stress (Shen et al., 2013), vascularization (Strieter, Burdick, Gomperts, Belperio, & Keane, 2005), and tumorigenesis (Jamieson et al., 2012). Moreover, the SNPs in CXCR2 gene have been reported to be associated with the risk of various diseases, such as acute pyelonephritis (Javor et al., 2012), autoimmune rheumatic diseases (Salim & Xavier, 2014), coronary atherosclerosis (Nasibullin et al., 2016), and prostate cancer (Franz et al., 2017).

Previous reports have noted that CXCR2 affects immune tolerance at the maternal fetal interface and decidual spiral artery remodeling, and therefore may contribute to preeclampsia and miscarriage (Kang et al., 2016; Pitman, Innes, Robson, Bulmer, & Lash, 2013). Furthermore, a recent study has demonstrated that the decreased CXCR2 in preeclamptic placentas may contribute to the development of preeclampsia through impairing trophoblast invasion by down‐regulating MMP‐2 and MMP‐9 via the Akt signaling pathway (Wu et al., 2016). These findings provide direct evidences that CXCR2 may be involved in the pathogenesis of preeclampsia. However, the role of CXCR2 gene polymorphism in the development of preeclampsia remains unexplored. Thus, we aimed to investigate the association between polymorphisms of CXCR2 gene and preeclampsia in Han Chinese women.

## 2 MATERIALS AND METHODS

### 2.1 Subjects

The case‐control study was approved by the ethics committee of West China Second University Hospital, Sichuan University. All the participants provided written informed consent. Totally 481 participants were recruited from West China Second University Hospital during September 2010 and March 2015. Preeclampsia was diagnosed as systolic blood pressure ≥140 mmHg and/or diastolic blood pressure ≥90 mmHg on at least two occasions after 20 weeks of gestation, association with proteinuria (>300 mg/24 hr and/or 1 + on dipstick testing) (Brown, Lindheimer, Swiet, Assche, & Moutquin, 2001). Severe preeclampsia was defined as systolic blood pressure ≥160 mmHg and/or diastolic blood pressure ≥110 mmHg, along with significant proteinuria (>2 g/24 hr and/or 2 + on dipstick testing) or evidence of multiorgan problems including pulmonary edema, seizures, oliguria, thrombocytopenia, liver dysfunction, or central nervous system perturbations (Brown et al., 2001). Preeclampsia was divided into early‐onset disease (<34 weeks) and late‐onset disease (≥34 weeks) （von Dadelszen, Magee, & Roberts, 2003）. A total of 238 preeclampsia patients ranging in age from 19 to 45 (mean ± *SD*, 30.26 ± 5.79) were divided into two groups, including 197 severe cases and 41 mild cases. And 243 normal pregnant women ranging in age from 18 to 42 (mean ± *SD*, 30.42 ± 4.89) were recruited, which uncomplicated with gestational hypertension and proteinuria during the same period.

Exclusion criteria included diabetes, heart diseases, chronic hypertension, kidney diseases, autoimmune diseases, thrombophilia, multiple pregnancy, and fetal malformation. Gestational age was confirmed by last menstrual period and/or routine ultrasound measurements in the first trimester.

### 2.2 DNA extraction and genotyping

Two SNPs (rs1126579 and rs2230054) in CXCR2 gene （NG_052975.1）were genotyped in the present study. Genomic DNA was extracted from 2 ml EDTA anticoagulated peripheral blood using DNA extraction kit (Bioteke, China) according to the instruction manual. Briefly, all PCR amplification systems were performed in a total volume of 25 μl, including 2.5 μl 10 × PCR buffer, 1.5 mmol/L MgCl_2_, 0.15 mmol/L dNTPs, 0.5 mmol/L each primer, 100 ng of genomic DNA, and 1U of Taq DNA polymerase. The investigated DNA sequences were amplified by the following primers: forward 5′‐CAAGATTCTAGCTATACATGGCTTG‐3′ and reverse 5′‐AAGAACGTGGCCTC GTCT‐3′ for rs1126579; forward 5′‐TACTCATCCAATGTTAGCCC‐3′ and reverse 5′‐CCAGGTTGTAGGGCAGCCTGC‐3′ for rs2230054.

The PCR conditions were initial denaturation at 94°C for 4 min, 33 circles for denaturation of 30 s at 94°C, followed by annealing 30 s at 57°C for rs1126579 and 60°C for rs2230054, with a final elongation at 72°C for 10 min. PCR products were digested by specific restriction enzymes (*ACCI*for rs1126579 and *PSTI*for rs2230054) at 37°C for overnight in a reaction volume of 10 μl.

### 2.3 Restriction fragment length polymorphism analysis

**Figure 1:**
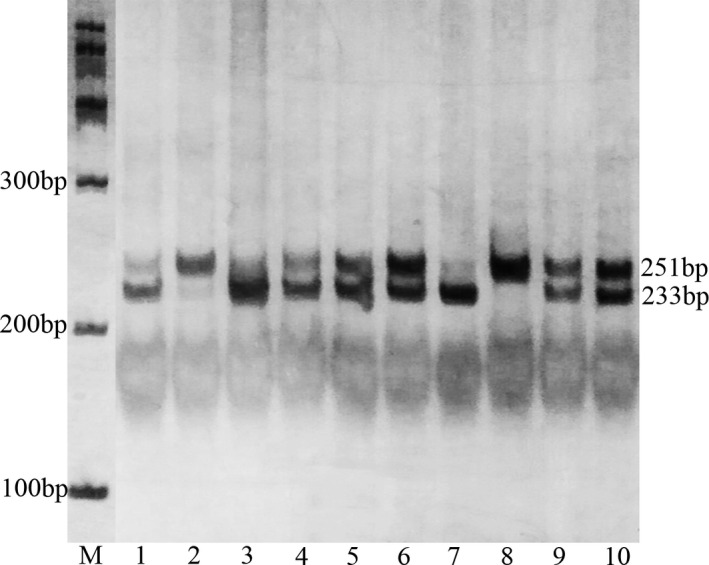
Polymerase chain reaction‐restriction fragment length polymorphism analysis of rs1126579 polymorphism of the CXCR2 gene. Lane M shows the size markers. 1, 3, 7: TT genotype (233 bp); 2, 8: CC genotype (251 bp); 4, 5, 6, 9, 10: TC genotype (251/233 bp)

**Figure 2:**
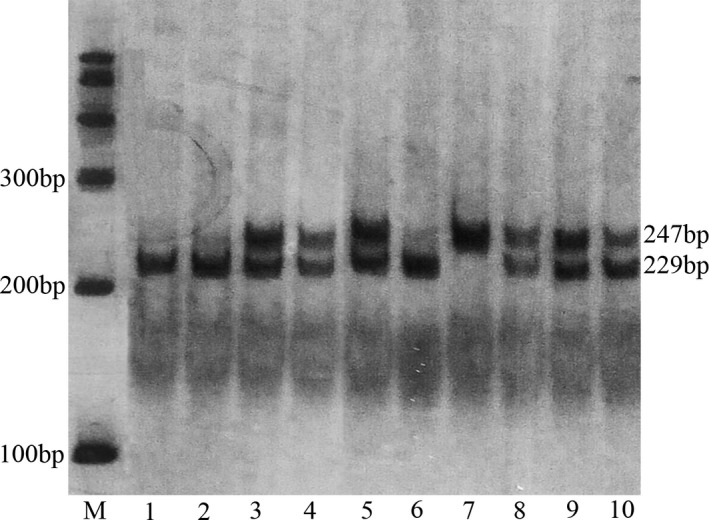
Polymerase chain reaction‐restriction fragment length polymorphism analysis of rs2230054 polymorphism of the CXCR2 gene. Lane M shows the size markers. 1, 2, 6: CC genotype (229 bp); 7: TT genotype (247 bp); 3, 4, 5, 8, 9, 10: TC genotype (247/229 bp)

Digested products were separated by a 6% polyacrylamide gel and stained with 1.5 g/L argent nitrate: for rs1126579, allele T is cuttable, emerging two fragments of 18 bp and 233 bp, allele C is uncuttable and the fragment is still 251 bp (Figure 1). For rs2230054, allele C is cuttable, yielding two fragments of 18 bp and 229 bp, allele T is uncuttable and the fragment is still 247 bp (Figure 2). For quality control, about 10% of the samples were randomly selected to perform repeated assays and the results were 100% concordant.

### 2.4 Statistical analysis

All data were analyzed using SPSS 19.0 (SPSS Inc, Chicago, IL). Allele and genotype frequencies of rs1126579 and rs2230054 polymorphisms were obtained by direct computing and the Pearson's chi‐square test was used to test Hardy–Weinberg equilibrium. Genotypic association tests in a case‐control pattern, assuming codominant, dominant, recessive, overdominant, or log‐additive genetic models were performed using SNPstats online software (www.snpstats.net/start.htm) (Sole, Guino, Valls, Iniesta, & Moreno, 2006). Chi‐square test was performed to evaluate the differences of the allele and genotype frequencies. Odds ratio (OR) and respective 95% confidence intervals were reported to evaluate the effects of any difference between alleles and genotypes. *p* values less than 0.05 were considered statistically significant.

## 3 RESULTS

The two entire SNPs in our study were successfully genotyped in 238 patients with preeclampsia and 243 control subjects. Genotype distributions of the two polymorphisms were consistent with Hardy–Weinberg equilibrium in control group (*p = *0.67 for rs1126579; *p* = 0.053 for rs2230054). As shown in Table 1, significantly increased risk of preeclampsia was observed to be associated with C allele of rs1126579 polymorphism (*p* = 0.03, OR = 1.48, 95% CI = 1.14–1.92).

**Table 1:** Allele frequencies of these two SNPs in women with and without preeclampsia

	Allele		OR (95% CI)	*p* Value
	T (%)	C (%)		
*rs1126579*				
Control	316 (65.0)	170 (35.0)		
PE	265 (55.7)	211 (44.3)	**1.48 (1.14–1.92)**	**0.03**
PE				
SPE	212 (53.8)	182 (46.2)	**1.60 (1.22–2.10)**	**0.001**
MPE	53 (64.6)	29 (35.4)	1.02 (0.62–1.66)	0.95
SPE				
Early‐onset	88 (53.0)	78 (47.0)	**1.65 (1.15–2.36)**	**0.006**
Late‐onset	124 (54.4)	104 (45.6)	**1.56 (1.32–2.15)**	**0.006**
*rs2230054*				
Control	134 (28)	352 (72)		
PE	147 (31)	329 (69)	1.17 (0.84–1.55)	0.26
PE				
SPE	122 (31.0)	272 (69.0)	1.18 (0.88–1.58)	0.27
MPE	25 (30.5)	57 (69.5)	1.15 (0.69–1.92)	0.58
SPE				
Early‐onset	51 (30.7)	115 (69.3)	1.17 (0.79–1.71)	0.44
Late‐onset	71 (31.1)	157 (68.9)	1.19 (0.84–1.68)	0.33

Note

Bold faced values indicate a significant difference at the 5% level.

PE: preeclampsia; MPE: mild preeclampsia; SPE: severe preeclampsia.

Women with preeclampsia were further divided into four subgroups: severe preeclampsia (197 cases), mild preeclampsia (41 cases), early‐onset severe preeclampsia (83 cases), and late‐onset preeclampsia (114 cases). Relative to the controls, an association with rs1126579 polymorphism was observed in women with severe preeclampsia (*p* = 0.03, OR* *= 1.48, 95% CI = 1.14–1.92), but not mild preeclampsia (*p* = 0.95, OR = 1.02, 95% CI = 0.62–1.66). Significantly increased risk of early‐onset preeclampsia was also identified to be associated with C allele of rs1126579 polymorphism (*p* = 0.006, OR = 1.65, 95% CI = 1.15–2.36), while the difference was also found between late‐onset preeclampsia and controls (*p* = 0.006, OR = 1.56, 95% CI = 1.32–2.15). However, our data indicated no significant difference in the allele frequencies of rs2230054 polymorphism between patients and control (Table 1).

As shown in Table 2, significantly increased risk of preeclampsia was found to be associated with the CC genotype of rs1126579 polymorphism in a codominant model, compared with TT genotype (*p* = 0.0039, OR = 2.11, 95%CI = 1.18–3.76). Compared with TT genotype, markedly increased risk of preeclampsia was associated with the TC/CC genotypes in a dominant model (*p* = 0.001, OR = 1.89, 95% CI = 1.29–2.78). Moreover, TC genotype carriers had a 1.48 fold increased risk of preeclampsia (95% CI = 1.04–2.12, *p* = 0.031) than that of TT/CC genotypes carriers. There were no significant differences observed between rs2230054 polymorphism and risk of preeclampsia (*p* > 0.05, Table 2).

**Table 2:** Genotype frequencies of SNPs in CXCR2 between patients and controls and their association with preeclampsia risk

Genetic model	Genotype	Patients	control	Logistic regression	
		*N* = 238 (%)	*N* = 243 (%)	OR (95% CI)	*p* Value
*rs1126579*					
Codominant	TT	65 (27.3%)	101 (41.6%)	1.00 (reference)	
	TC	135 (56.7%)	114 (46.9%)	1.84 (1.23–2.74)	
	CC	38 (16%)	28 (11.5%)	**2.11 (1.18–3.76)**	**0.0039**
Dominant	TT	65 (27.3%)	101 (41.6%)	1.00 (reference)	
	TC/CC	173 (72.7%)	142 (58.4%)	**1.89 (1.29–2.78)**	**0.001**
Recessive	TT/TC	200 (84%)	215 (88.5%)	1.00 (reference)	
	CC	38 (16%)	28 (11.5%)	1.46 (0.86–2.47)	0.16
Overdominant	TT/CC	103 (43.3%)	129 (53.1%)	1.00 (reference)	
	TC	135 (56.7%)	114 (46.9%)	**1.48 (1.04–2.12)**	**0.031**
Log‐additive	—	—	—	1.54 (1.17–2.03)	**0.0019**
*rs2230054*					
Codominant	CC	101 (42.4%)	121 (49.8%)	1.00 (reference)	
	TC	127 (53.4%)	110 (45.3%)	1.38 (0.96–2.00)	
	TT	10 (4.2%)	12 (4.9%)	1.00 (0.42–2.41)	0.21
Dominant	CC	101 (42.4%)	121 (49.8%)	1.00 (reference)	
	TC/TT	137 (57.6%)	122 (50.2%)	1.35 (0.94–1.93)	0.11
Recessive	CC/TC	228 (95.8%)	231 (95.1%)	1.00 (reference)	
	TT	10 (4.2%)	12 (4.9%)	0.84 (0.36–1.99)	0.7
Overdominant	CC/TT	111 (46.6%)	133 (54.7%)	1.00 (reference)	
	TC	127 (53.4%)	110 (45.3%)	1.38 (0.97–1.98)	0.076
Log‐additive	—	—	—	1.22 (0.89–1.66)	0.21

Note

Bold faced values indicate a significant difference at the 5% level.

CXCR2 gene version number: NG_052975.1.

As shown in Table 3, significant differences were identified between severe preeclampsia and the controls under dominant (*p* < 0.01) and overdominant (*p* = 0.022) model. Similar results were observed in the late‐onset group under dominant (*p* < 0.01), and overdominant (*p* = 0.022) models. Significantly increased risk of early‐onset preeclampsia was also identified to be associated with the TC/CC genotypes in a recessive model (*p* = 0.013, OR = 1.97, 95% CI = 1.14–3.42) compared with the TT genotype. However, no significant association in genotype frequencies of rs2230054 polymorphism has been detected between patients and control (*p* > 0.05, Table 4).

**Table 3:** Genotype frequencies of rs1126579 in women with and without preeclampsia

Rs1126579									
	Genotype			Genetic model					
				Dominant		Recessive		Overdominant	
				TT VS TC/CC		TT/TC VS CC		TT/CC VS TC	
	TT	TC	CC	OR (95% CI)	*p* Value	OR (95% CI)	*p*Value	OR (95% CI)	*p* Value
PE									
SPE	49 (24.9%)	114 (57.9%)	34 (17.3%)	**2.15 (1.42–3.24)**	**<0.01**	1.60 (0.93–2.75)	0.086	**1.55 (1.06–2.27)**	**0.022**
MPE	16 (39.0%)	21 (51.2%)	4 (9.8%)	1.11 (0.56–2.19)	0.76	0.83 (0.28–2.50)	0.74	1.19 (0.61–2.30)	0.61
SPE									
Early‐onset	22 (26.5%)	44 (53.0%)	17 (20.5%)	**1.97 (1.14–3.42)**	**0.013**	**1.98 (1.02–3.84)**	**0.049**	1.28 (0.77–2.10)	0.34
Late‐onset	27 (23.7%)	70 (61.4%)	17 (11.9%)	**2.29 (1.39–3.78)**	**<0.01**	1.35 (0.70–2.57)	0.37	**1.80 (1.14–2.83)**	**0.01**
Control	101 (41.6%)	114 (46.9%)	28 (11.5%)						

Note

PE: preeclampsia; MPE: mild preeclampsia; SPE: severe preeclampsia.

Bold faced values indicate a significant difference at the 5% level.

**Table 4:** Genotype frequencies of rs2230054 in women with and without preeclampsia

rs2230054									
	Genotype			Genetic model					
				Dominant		Recessive		Overdominant	
				CC VS TC/TT		CC/TC VS TT		CC/TT VS TC	
	CC	TC	TT	OR (95% CI)	*p* Value	OR (95% CI)	*p*Value	OR (95% CI)	*p* Value
PE									
SPE	84 (42.6%)	104 (52. 8%)	9 (4.6%)	1.33 (0.91–1.95)	0.13	0.92 (0.38–2.23)	0.86	1.35 (0.93–1.97)	0.12
MPE	17 (41.5%)	23 (56.1%)	1 (2.4%)	1.40 (0.72–2.74)	0.32	0.48 (0.06–3.80)	0.44	1.54 (0.79–3.01)	0.2
SPE									
Early‐onset	35 (42.2%)	45 (54.2%)	3 (3.6%)	1.36 (0.82–2.25)	0.23	0.72 (0.20–2.62)	0.61	1.43 (0.87–2.36)	0.16
Late‐onset	49 (43.0%)	59 (51.8%)	6 (5.2%)	1.32 (0.84–2.06)	0.23	1.07 (0.39–2.93)	0.9	1.30 (0.83–2.03)	0.25
Control	121 (49.8%)	110 (45.3%)	12 (4.9%)						

PE: preeclampsia; MPE: mild preeclampsia; SPE: severe preeclampsia.

## 4 DISCUSSION

Preeclampsia is a complex pregnancy‐specific hypertensive syndrome and poses a serious threat to maternal and fetal health. Genetic factors are believed to be involved in the development of preeclampsia (Chesley & Cooper, 1986; Morgan, 2013). CXCR2, the receptor of the CXC chemokines, is identified to contribute to the pathogenesis of preeclampsia. However, little is known about the possible impact of CXCR2 gene polymorphism on the development of preeclampsia.

Our study aimed to analyze the influence of CXCR2 polymorphisms on the predisposition of preeclampsia. Two possible variation sites of CXCR2 were identified, including one 3′ untranslated region (rs1126579) and one exon (rs2230054) sequence. The rs1126579 polymorphism has been reported to be associated with the susceptibility of lung cancer (Wang et al., 2017), septic shock (Cardoso et al., 2015), colorectal cancer (Slattery & Lundgreen, 2014), and biliary tract cancers and stones (Hsing et al., 2008), and the rs2230054 polymorphism has been reported to be associated with the risk for colorectal cancer (Gerger et al., 2011) and biliary tract cancers and stones (Hsing et al., 2008). To the best of our knowledge, no studies have examined the role of CXCR2 polymorphisms in the development of preeclampsia before.

Our study found that significantly increased risk of preeclampsia was identified to be associated with C allele of rs1126579 polymorphism. We provided primary evidences that CC and TC/CC genotypes (compared with TT genotypes) of rs1126579 polymorphism were more frequent in patients with severe preeclampsia. Moreover, TC/CC genotypes (compared with TT genotype) and TC genotype (compared with TT/CC genotypes) were more frequent in patients with severe preeclampsia or late onset severe preeclampsia. Similar, TC/CC genotypes (compared with TT genotype) were more frequent in patients with early onset severe preeclampsia. These findings suggested that rs1126579 polymorphism turned out to be a risk factor for severe preeclampsia, which has not been reported before. Furthermore, previous studies demonstrated that CC genotype carriers of rs1126579 polymorphism were associated with increased risk of lung cancer in both European American and Japanese populations (Ryan et al., 2015) and biliary tract cancers and stones in Chinese population (Hsing et al., 2008). Cardoso et al. (2015) also proposed that the C allele of rs1126579 polymorphism was found to be associated with higher risk of septic shock. However, some studies suggested that CC genotype carriers of rs1126579 polymorphism displayed decreased risk of colorectal cancer (Slattery & Lundgreen, 2014; Bondurant et al., 2013), hepatitis C virus infection (Zang et al., 2018), and stroke (Timasheva, Nasibullin, & Mustafina, 2015). Ni et al. （^2017^） showed that rs1126579 polymorphism was not associated with periodontitis susceptibility in Chinese population. These inconsistent findings are possibly due to ethnic difference and different types of diseases. Further studies are necessary to verify this genetic association.

Our study did not show any differences in the allele and genotype frequencies of the rs2230054 polymorphism between patients and controls. Current researches on the relationship between rs2230054 polymorphism and diseases are controversial. Our data were in agreement with the studies reporting that rs2230054 polymorphism was not associated with susceptibility of periodontitis in Chinese population and prosthetic joint infection in Czech population （Ni et al., 2017; Navratilova, Gallo, Mrazek, & Petrek, 2012）. However, Hsing et al. (2008) discovered that TT genotype carriers of rs2230054 polymorphism showed increased risk of biliary tract cancers and stones in Chinese population. Some reasons referring ethnic difference and different types of diseases may contribute to the inconsistent results. Further investigation is required.

We should point out that one strength of the present study is its genetic homogeneity of the study population, including individuals only from the Sichuan province with the same genetic background of Han Chinese. However, some limitations deserve our consideration. We were unable to measure the plasma CXCR2 protein levels for its nonsecretion into the blood circulation. Hence, there is a lack of direct biochemical/functional evidence of CXCR2 polymorphism with altered protein levels. No genotyping was performed in the placentas of these participants. Besides, the relatively small study sample size should be recognized. Thus, further investigations are warranted to complement our results and illuminate the relationship between CXCR2 gene variation and the development of preeclampsia.

In conclusion, we firstly described the genetic association between rs1126579 polymorphism in CXCR2 gene and increased risk of preeclampsia, while there is no association between rs2230054 polymorphism and the disorder. Whether the rs1126579 variation has a possible link with potential changes in the function of CXCR2‐associated pathways in patients with preeclampsia remains to be explored in the future.

## CONFLICT OF INTEREST

The authors declare that they have no competing interests.
